# Visual Sorting of Express Parcels Based on Multi-Task Deep Learning

**DOI:** 10.3390/s20236785

**Published:** 2020-11-27

**Authors:** Song Han, Xiaoping Liu, Xing Han, Gang Wang, Shaobo Wu

**Affiliations:** Automation School, Beijing University of Posts and Telecommunications, Beijing 100876, China; hansong@bupt.edu.cn (S.H.); hax@bupt.edu.cn (X.H.); wg58977@163.com (G.W.); wushaobo@bupt.edu.cn (S.W.)

**Keywords:** robotic sorting, object detection network, multi-task deep learning, intelligent logistics sorting system, warehouse automation

## Abstract

Visual sorting of express parcels in complex scenes has always been a key issue in intelligent logistics sorting systems. With existing methods, it is still difficult to achieve fast and accurate sorting of disorderly stacked parcels. In order to achieve accurate detection and efficient sorting of disorderly stacked express parcels, we propose a robot sorting method based on multi-task deep learning. Firstly, a lightweight object detection network model is proposed to improve the real-time performance of the system. A scale variable and the joint weights of the network are used to sparsify the model and automatically identify unimportant channels. Pruning strategies are used to reduce the model size and increase the speed of detection without losing accuracy. Then, an optimal sorting position and pose estimation network model based on multi-task deep learning is proposed. Using an end-to-end network structure, the optimal sorting positions and poses of express parcels are estimated in real time by combining pose and position information for joint training. It is proved that this model can further improve the sorting accuracy. Finally, the accuracy and real-time performance of this method are verified by robotic sorting experiments.

## 1. Introduction

With the prosperity of the e-commerce industry, the rapid development of the logistics industry has given rise to massive orders for logistics companies, which has brought a heavy load for the sorting work of logistics systems. At this stage, traditional manual sorting can no longer cope with such sorting loads. Therefore, vision-based intelligent logistics sorting systems have significant research value. Detecting objects’ positions, categories, and attitude information accurately and quickly has become a key issue in the implementation of intelligent sorting systems.

Earlier research on multi-object sorting mostly used sorting methods based on the geometric modeling of objects, which realized the picking up or emptying of the objects in the boxes in two steps. These methods [1,2,3,4] first use object detection algorithms to detect the position of the object, and then use the known two-dimensional or three-dimensional model to estimate the pose of the object. For example, Schwarz et al. [4] proposed a sorting system in the Amazon competition in 2016, in which the image information of a dual-depth camera is merged to eliminate the image gap caused by the perspective of a single camera. Then, two deep learning methods are used for object detection and semantic segmentation, and an ICP-based (iterative closest point) [5] object model matching algorithm is used to estimate the pose of the object. These methods require a two-dimensional or three-dimensional model of each item. Therefore, the workload of preliminary preparation is large, and the computation capacity usually makes it difficult to meet the real-time requirements of practical applications.

In the research on robot sorting methods in recent years, researchers have mostly used deep neural networks to directly detect the grasping positions and poses. Some of those use grasping rectangles to represent the positions and poses of grasping end-effectors, and deep learning networks are used to directly detect those positions and poses. Kumra et al. [6] proposed a multi-modal detection model based on deep learning for object grasping detection using color and depth information, in which a five-dimensional oriented rectangle representation is used to describe the grasping, i.e., (x,y,w,h,θ), where (x,y) represents the grasping coordinates, *w* and *h* respectively represent the required opening distance and length of the parallel gripper, and θ represents the tilt angle of the object. The authors trained and tested on the Cornell dataset and achieved an accuracy of 89.21% while ensuring the real-time performance of the system. A similar oriented rectangle representation was also used in [7,8,9,10]. Zhang et al. [10] proposed a fully convolutional network for grasping area detection based on a directed anchor, which realizes real-time detection of the object grasping position and obtains a high accuracy on the Cornell grasp dataset. However, the object poses of most of the above models only have one degree of freedom, which is only suitable for cases in which there is not much inclination of the object and the two-dimensional image plane can be mapped well into three-dimensional space. Thus, it is difficult to accurately detect when the objects are heavily stacked.

In another type of research, deep learning models based on segmentation have been used to detect the grasping areas. As the winning team in the Amazon sorting competition in 2017, Zeng et al. [11] proposed a robotic sorting system for known and new objects in complex environments. Using multiple installed RGB-D cameras as data sources, this system exploits deep neural networks based on ResNet-101 [12] and Fully Convolutional Networks (FCN) [13] to segment pixel-level grasping candidate regions, and then adopts the regression score and a series of optimization strategies to select the best grasping point. The system can achieve a grasping accuracy of 96.7% with a gripper and 92.4% with a sucker, and it can sort new objects well. Similarly, Nguyen et al. [14] used two deep neural networks connected in series, one as an object detector and another to detect the functional area of the object. It was proved that the combination of an object detector and a post-optimization using dense conditional random fields (CRF) [15] could achieve a high detection effect, which was experimentally verified on a full-size humanoid robot. On this basis, Thanh-Toan Do et al. [16] proposed AffordanceNet, which borrows the idea of the popular instance segmentation algorithm Mask R-CNN [17]. An end-to-end network structure is used to implement object classification and the segmentation of functional areas. Shao et al. [18] combined ResNet with the U-net [19] structure, a special framework of convolution neural networks (CNNs), to predict the picking region without recognition and pose estimation, which makes the robotic picking system learn picking skills from scratch. This type of system more directly determines the grasping points or candidate grasping areas. However, image segmentation algorithms often require heavy network structures; thus, the accuracy and the real-time performance in industrial applications have to be weighed.

The sorting research for express parcels in this paper is applied to the real logistics warehousing environment. It not only needs to accurately detect parcels with different sizes and partial occlusion, but also needs to ensure the efficiency and timeliness of the system. Thus, the detection methods based on object modeling are not applicable. Moreover, unlike the daily necessities in the existing datasets, the confusing colors of the surfaces and the stacking on top of each other make the grasping areas of the parcels lack prominent features. It is necessary to establish a more reasonable model for the objects’ grasping points in three-dimensional environments. Aiming at the issue of fast parcel sorting, Han et al. [20] proposed a robot picking method based on deep neural networks in complex scenes. The multi-layer shallow feature map and the final feature map are merged to extract more detailed candidate grasping areas, and then a cascaded convolutional optimal grasping position detection network based on key points is used to achieve real-time estimation for target objects. The grasping point model of this research is designed in three-dimensional form. The position and attitude of the object can be estimated from the key points using the above method, but there is still room for improvement in detection accuracy. At the same time, the edge devices in the logistics industry have small memories, weak computing performance, and insufficient network inference capabilities. A lighter network structure is required to meet the real-time requirements for industry scenes.

To overcome the aforementioned challenges, in this paper, we propose a rapid sorting method based on multi-task deep learning. First, a new lightweight network model architecture is proposed to improve detection speed of stacked parcels without loss of accuracy. Second, a multi-task optimal grasping position estimation network model based on key points is proposed to predict the optimal grasping position of express packages in real time. Finally, the optimal grasping point and depth information of the scene are used to determine the pose of the target object to realize automatic grasping of parcels with an industry robot.

The remainder of this paper is organized as follows: Section 2 represents the overall framework of this parcel sorting method and then demonstrates the lightweight object detection network and multi-task optimal sorting network in detail. Section 3 presents experiments to verify the correctness and efficiency of the algorithm. The conclusion and future work are presented in Section 4.

## 2. Sorting Method for Express Parcels in Complex Scenes

### 2.1. Overall Framework

**Figure 1:**
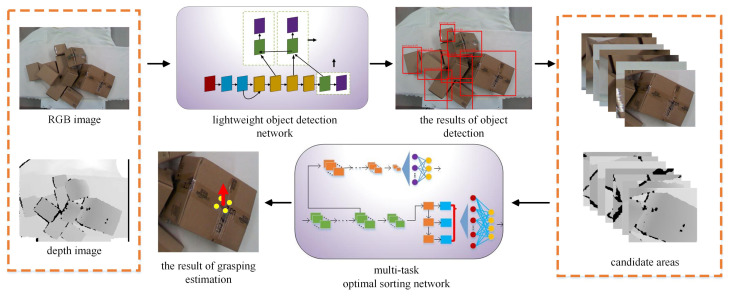
The overall framework of a parcel sorting method based on multi-task deep learning. A lightweight object detection algorithm is used to sense the environment, and the position and category of the express parcels are quickly and accurately obtained. An optimal sorting position and pose estimation network model based on multi-task deep learning are used to estimate the location and attitude for the grasping of the parcels.

The overall framework of a parcel sorting method based on multi-task deep learning is shown in Figure 1. In the process of separating stacked parcels, a lightweight object detection algorithm is used to sense the environment, and the position and category of the express parcels are quickly and accurately obtained. Then, an optimal sorting position and pose estimation network model based on multi-task deep learning are used to estimate the location and attitude for the grasping of the parcels. In Figure 1, the yellow dots indicate the detected key points, and the red arrow represents the grasping action, the end of which indicates the grasping position.

### 2.2. Lightweight Object Detection Network

In recent years, large-scale datasets, high-end modern GPUs, and new network architectures have allowed the development of unprecedentedly large CNN models. Both two-stage models, such as Fast R-CNN [21] and Faster R-CNN [22], and end-to-end models, such as YOLO [23] and SSD [24], often have heavy network structures to ensure the desired accuracy in object detection tasks. Among them, the YOLOv3 [25] object detection network model has been widely used in many industrial fields due to its excellent performance in speed and accuracy. However, considering the higher real-time requirements and more limited equipment performance in an intelligent logistics sorting system, the inference speed of YOLOv3 is far from enough. On the other hand, although high detection accuracy is required in the express parcel sorting scenes, there are still a large number of redundant parameters that can be pruned from YOLOv3 for use in the task of express parcel sorting. Therefore, in order to apply the object detection model to the intelligent logistics sorting system better, we propose a pruning strategy for YOLOv3 to obtain a lightweight object detection model with no accuracy loss.

In the previous works [26,27,28], networks were thinned by pruning strategies to reduce the weight of the network. Network sparsification can be implemented in different aspects, such as with the parameters, kernels, channels, and layers. Fine-grained-level (e.g., weight-level) sparsity gives the highest flexibility and generality, which will lead to a higher rate of compression, but it usually requires special software or hardware accelerators for fast inference on the sparse model [29]. On the contrary, the coarsest-layer-level sparsity does not require special packages to speed up the inference. However, when some network layers are pruned, the network lacks the corresponding flexibility. In comparison, channel-level sparsity provides a nice tradeoff between flexibility and ease of implementation. It can be applied to any typical CNNs or fully connected neural networks, and more lightweight network models can be obtained, which can enable efficient inference and lower memory usage.

For judging the importance of the network channel, we introduce a scale variable γ for each channel to evaluate its importance. Then, the training weights and scale variables are applied to the subsequent sparse training. Finally, the network is pruned according to the scale variable. The loss function of the network model that joins weights and scale variables for training is

(1) $$L=\sum_{\left(x,y\right)}l\left(f\left(x,W\right),y\right)+\lambda_{d}\sum_{\gamma \in \Gamma }g\left(\gamma \right),$$

where (x,y) denotes the training input and target, *W* denotes the trainable weights, $\sum_{\left(x,y\right)}l\left(f\left(x,W\right),y\right)$ corresponds to the normal training loss of a CNN, and g(·) is a sparsity-induced penalty on the scaling factors. The L1-norm represents the sum of the absolute values of the elements in the vector, and L1 regularization has a property that can generate a sparse model, so we use g(s)=|s| to sparsify the network. $\lambda_{d}$ is a dynamic weight used to balance the relationship between the convolutional neural network and the sparse network.

We set the initial and final weight as $\lambda_{s}$ and $\lambda_{f}$, respectively. We use a cosine function to make $\lambda_{d}$ dynamically adjust as the number of network training iterations increases, as shown in Equation (2). $I_{cur}$ and $I_{max}$ represent the current number of iterations and the maximum number of iterations for network training, respectively. In the initial stage of network training, g(·) accounts for a larger proportion of losses, which can guarantee greater sparseness of the network. It is more conducive to judging the importance of the channel. In the later stage, as $\lambda_{d}$ decreases, the sparseness slows down, but better detection accuracy can be guaranteed. Compared with a fixed weight, dynamical weight can make the network’s sparseness and detection accuracy a reasonable trade-off.

(2) $$\lambda_{d}=\lambda_{f}+\left(\lambda_{s}-\lambda_{f}\right)\cos\left(\frac{I_{cur}}{I_{max}}\cdot \frac{\pi }{2}\right)$$

When the network model is in the process of joint training, the scale variable γ can autonomously guide the network to identify importance of each channel, and it can safely remove the channel without seriously affecting the network performance. The scale variable is determined by batch normalization (BN). By designing a simple and effective method that includes channel scale variables and normalizing the batch input data, BN can make the network quickly converge and achieve better representation capabilities. Let $z_{in}$ and $z_{out}$ be the input and output of a BN layer; $\mu_{B}$ and $\sigma_{B}$ denote the mean and standard deviation values of input activations over the current mini-batch, and $z_{mid}$ denotes the intermediate result after the first operation. The BN layer performs the following transformation:

(3) $$z_{mid}=\frac{z_{in}-\mu_{B}}{\sqrt{\sigma_{B}^{2}+\epsilon }}$$

(4) $$z_{out}=\gamma z_{mid}+\beta ,$$

where γ and β are trainable scale and shift transformation parameters, which can recover the original activations to ensure the representation ability of the networks. ϵ is set to prevent the instability caused by data overflow, and has a certain effect of smoothing and denoising.

**Figure 2:**
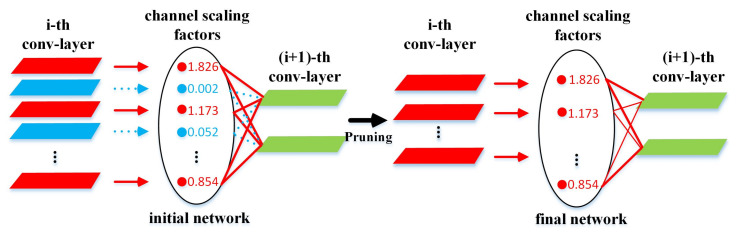
Channel-level pruning.

In the process of guiding channel-level sparsification, we can obtain a thinning model, in which many BN scale variables are close to zero. Then, those channels close to 0 are pruned according to a global threshold. In the pruning process, it is important to remove the input and output terms of the corresponding channel. As shown in Figure 2, a more lightweight model structure can be directly obtained without additional software or hardware resources for sparsification.

**Figure 3:**
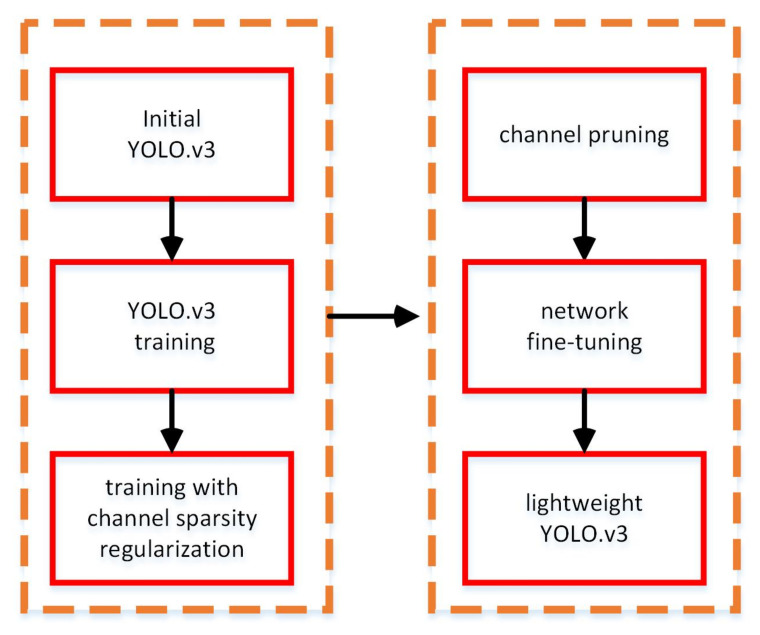
Flowchart of the network pruning procedure.

Although a more lightweight model is obtained with lower running memory requirements and faster running speed, pruning a certain number of channels will cause the model’s representation ability to decrease significantly. Therefore, it is necessary to compensate through subsequent fine-tuning training of the model. After experiments, it was validated that the pruned YOLOv3 network model can achieve as considerable of an accuracy as before. The flowchart of the acquisition process of the lightweight network is shown in Figure 3.

**Figure 4:**
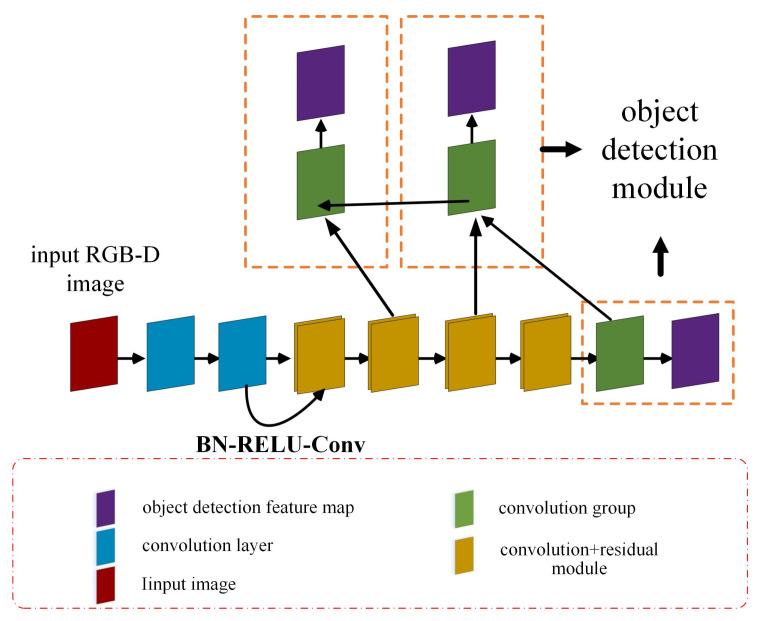
Structure of the lightweight detection network.

The YOLOv3 network model was processed through the pruning strategies above, and the method in [20] was used to fuse the channels of multi-layer shallow feature maps and deep feature maps to extract more detailed features, which improves the speed and accuracy of the detection. The model structure is shown in Figure 4.

Firstly, down-sampling is performed on the shallow feature maps. Then, we propose a BN–rectified linear unit (RELU)–convolution layer (Conv) module consisting of batch normalizations, activating linear units (RELUs, rectified linear units), and convolution layers (Conv) to conduct the information fusion. It was designed to gain more detailed information and more delicate features of objects, and to prevent overfitting caused by having too many channels after channel fusion. Subsequently, the structure is designed similarly to a spatial pyramid, which detects multi-scale feature maps for target objects of different sizes. The sizes of the three target detection feature maps and their corresponding preset bounding boxes are shown in Table 1 according to the clustering of the COCO dataset.

### 2.3. Multi-Task Optimal Sorting Position and Pose Estimation Network

**Figure 5:**
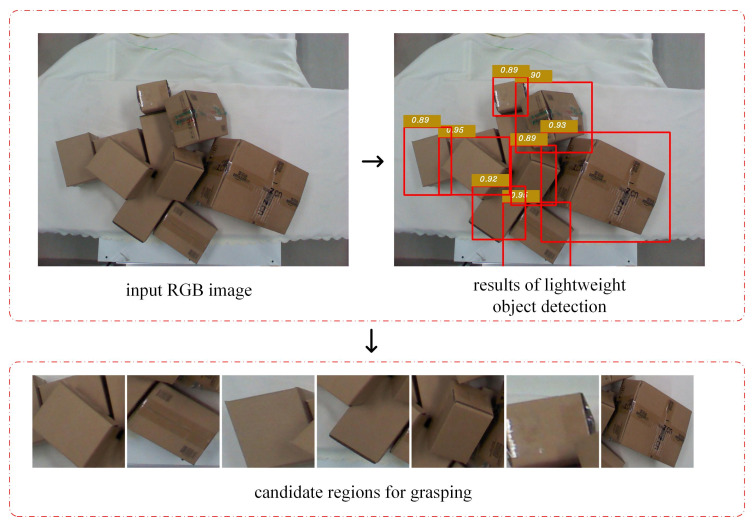
Candidate regions for grasping after lightweight object detection.

Before determining the position and pose of grasping, object detection was performed using the lightweight model proposed above to obtain candidate regions for grasping within the field of view of the camera. The detection results are shown in Figure 5.

**Figure 6:**
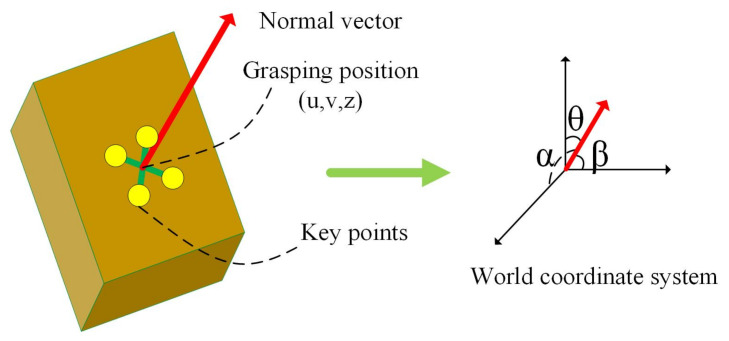
The model of the optimal sorting positions and poses for express parcels.

Accurately and efficiently obtaining the optimal sorting positions and poses of the express parcels is the key for robots to complete the sorting task. In order to achieve this goal, a multi-task deep learning network model based on key points is proposed, which can accurately detect the optimal sorting positions and poses in real time in a complex environment with disorderly stacking and mutual blocking. This paper uses a similar method to that of [20] to model the optimal sorting positions of the packages in three dimensions. As shown in Figure 6, the cuboid represents the express package to be picked. Four key points are used to characterize the optimal picking area, and their intersections are used as the final optimal sorting position. The sorting vector is represented as (u,v,z,α,β,θ), where (u,v,z) is the robot grasping position in the world coordinate system, and (α,β,θ) is used to characterize the three-dimensional posture of the object to be picked.

**Figure 7:**
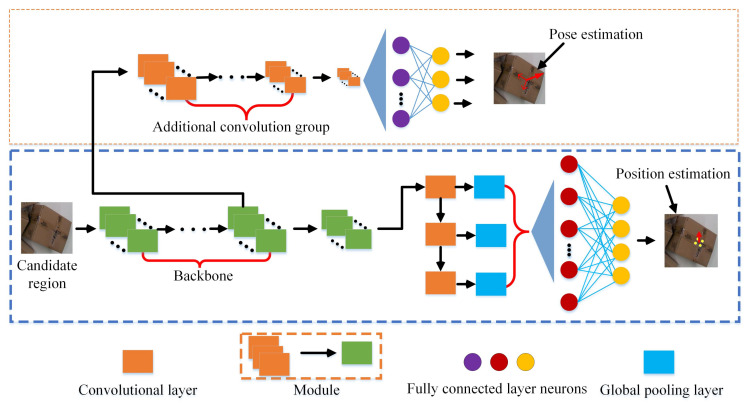
Optimal multi-task sorting position and pose estimation network model.

Unlike the method in [20], the network model in this paper is an end-to-end structure. The overall framework of the network is shown in Figure 7. In the training phase, the network first predicts the four key points of the optimal sorting surface of the sample. At the same time, pose information is exploited to constrain the positions of the key points, making it possible to determine the positions of the key points more efficiently and accurately. Finally, the intersection of key points is calculated as the sorting position, that is, the robot’s grasping position. The coordinates of the grab position in the world coordinate system are (u,v,z), as shown in Figure 6. The pose of the robot’s grasping is estimated by subsequent accurate calculations.

The model’s representation ability basically depends on the design of the loss function, especially when the training dataset is not sufficient enough. We use $\mathrm{gts}:=[x_{1}^{T},y_{1}^{T},x_{2}^{T},y_{2}^{T},x_{3}^{T},y_{3}^{T},x_{4}^{T},y_{4}^{T}]$ to represent the ground truth of the four key points and $\mathrm{pres}:=[x_{1}^{P},y_{1}^{P},x_{2}^{P},y_{2}^{P},x_{3}^{P},y_{3}^{P},x_{4}^{P},y_{4}^{P}]$ to represent the predicted key points. We usually directly use mean square error or root mean square error to design the loss function, but they are not reliable enough on average to measure the key points of different parts without considering geometric information. For example, $\left(x_{i}^{T},y_{i}^{T}\right),\left(x_{i}^{P},y_{i}^{P}\right)$ give the true and predicted coordinates of a key point in the image. Their deviation is $d=\sqrt{{\left(x_{i}^{T}-x_{i}^{P}\right)}^{2}+{\left(y_{i}^{T}-y_{i}^{P}\right)}^{2}}$. If two pairs of points are mapped from 3D to a 2D image, the distance in the 3D scene may be very different. Therefore, integrating attitude information into the loss function will be more helpful for improving the accuracy of key point positioning. For express parcels, in the complex environment of disorderly stacking and mutual obstruction, the pose of each express parcel is different, and the direction in which the robot grasps is one of the normal vector directions that can represent the pose. Therefore, we optimize the positions of key points by fusing the pose information and key point information, and take the error correction of key point correlation into account to obtain more accurate estimation results. The form of the loss function is as follows:

(5) $$LOSS=E_{pose}+E_{position}$$

(6) $$E_{pose}=\sqrt{\frac{1}{3}\left({\left(\alpha^{T}-\alpha^{P}\right)}^{2}+{\left(\beta^{T}-\beta^{P}\right)}^{2}+{\left(\theta^{T}-\theta^{P}\right)}^{2}\right)}$$

(7) $$E_{position}=\sqrt{\frac{1}{n}\sum_{i=1}^{n}Li+\lambda_{L}\left(L_{Slope}^{2}+L_{Parallel}^{2}\right)}$$

(8) $$L_{i}=\sqrt{{\left(x_{i}^{T}-x_{i}^{P}\right)}^{2}+{\left(y_{i}^{T}-y_{i}^{P}\right)}^{2}}$$

(9) $$L_{Slope}=\sqrt{{\left(\frac{y_{2}^{T}-y_{1}^{T}}{x_{2}^{T}-x_{1}^{T}}-\frac{y_{2}^{P}-y_{1}^{P}}{x_{2}^{P}-x_{1}^{P}}\right)}^{2}}$$

(10) $$L_{Parallel}=\sqrt{{\left(\frac{y_{2}^{P}-y_{1}^{P}}{x_{2}^{P}-x_{1}^{P}}-\frac{y_{3}^{P}-y_{4}^{P}}{x_{3}^{P}-x_{4}^{P}}\right)}^{2}},$$

where ${\left(\cdot \right)}^{T}$ and ${\left(\cdot \right)}^{P}$ represent the true value and the predicted value, respectively. $E_{pose}$ and $E_{position}$ denote the loss of the pose and the loss of the position, respectively. $L_{i}$ represents the loss of the key point, i(i=1,2,3,4). $\lambda_{L}$ denotes a weight to balance the two parts in $E_{position}$. $L_{Slope}$ indicates the correlation between the positions reflected by the predicted value and the real value. $L_{Parallel}$ represents the loss of the positional relationship between the four predicted key points.

From the above loss function, it can be intuitively seen that the fusion of three-dimensional attitude deviation is more reasonable compared to using only two-dimensional position deviation, which can help improve the accuracy and robustness of the system. The network is an end-to-end structure instead of a cascaded network [20], which simplifies the training process and can effectively improve the training speed.

Using the multi-task optimal picking pose estimation algorithm based on key points, the optimal sorting position for each object can be obtained, which is the grasping point of the robot. In our research, the pose output in the algorithm is used to help fine-tune key point detection. After we have the position, a method to calculate the pose is used to obtain the sorting pose of the parcels combined with depth information. Then, the position and the pose of grasping are transformed into a vector in the coordinate system of the robot base through a coordinate transformation, that is, (u,v,z,α,β,θ).

After predicting the four key points of the picking area, three key points can be selected to determine the plane to be sorted by a robot. The attitude of the end effector of the robot is perpendicular to the plane, that is, a normal vector of the plane, which can represent the pose of the parcel. Generally, vacuum suction is used as the end effector of a robot to suck box-shaped parcels. The opposite direction of the normal vector obtained is the direction of the end effector of the robot. The attitude of the end effector can be calculated as

(11) $$n=P_{1}P_{2}\times P_{1}P_{3}=\left(\begin{array}{ccc} i & j & k\\ x_{2}-x_{1} & y_{2}-y_{1} & z_{2}-z_{1}\\ x_{3}-x_{1} & y_{3}-y_{1} & z_{3}-z_{1}, \end{array}\right)$$

where $P_{1}$ and $P_{2}$ are the two direction vectors of the plane, respectively.

## 3. Experiment and Analysis

**Figure 8:**
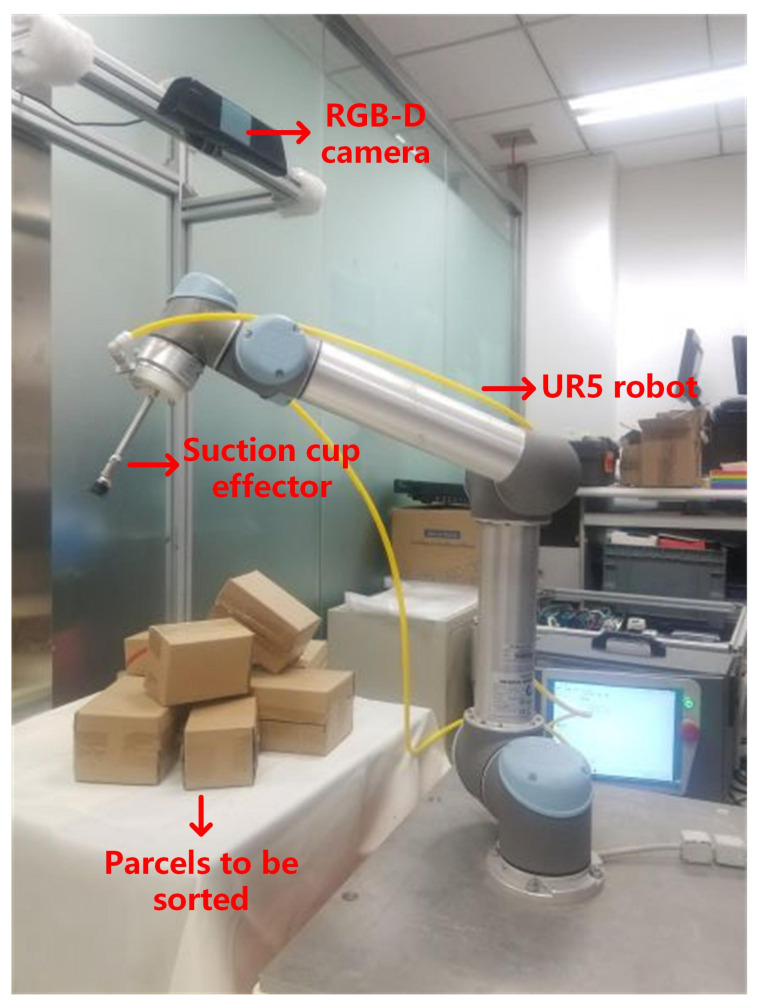
Vision-based robot sorting experiment platform.

During the training and testing processes of object detection and optimal sorting estimation, we conducted experiments using a GTX1080TI GPU with 11 GB, 32 GB memory, and an i7 8700k, six-core 12-thread CPU. The deep learning frameworks used for training were Tensorflow1.3.0 and Keras 2.1.0. In order to simulate the actual parcel sorting scenes in our laboratory and to verify the correctness and effectiveness of the algorithm, an RGB-D vision-based robot sorting experiment platform was set up, which mainly included a UR-5 six-degree-of-freedom robot, an RGB-D deep vision sensor (Kinect V2), a suction cup effector, etc. As shown in Figure 8, the objects to be sorted were box-shaped express parcels with different sizes that were randomly placed within the RGB-D camera’s field of view.

### 3.1. Experiment on Object Detection

For training the lightweight object detection model, the YOLOv3 initial model was trained at first, and then a thinning training was performed. The data for this experiment came from the RGB-D camera. The training set had 2140 images, the validation set had 400 images, and the test set had 400 images. The hyperparameters required for model training are shown in Table 2. In our experiment, the base learning rate was set to 0.01 and divided by 10 at iterations 2000 and 4000, respectively.

**Figure 9:**
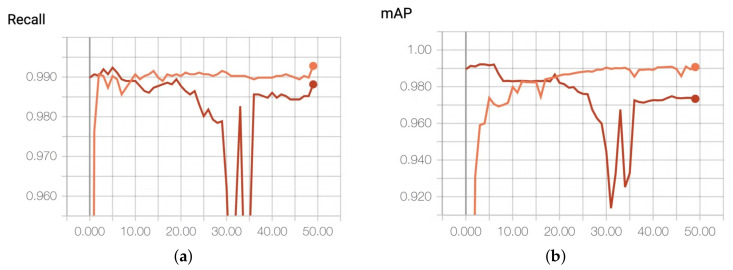
Average recall (AR) and mean average precision (MAP) during normal training and sparse training. (**a**) AR curves. (**b**) MAP curves.

The changes in average recall (AR) and mean average precision (MAP) during training are shown in Figure 9. In these two figures, the abscissas both represent the epochs of training, and the ordinate represents the AR and MAP of the network, where orange and positive red curves represent normal training and sparse training, respectively. It can be observed that the accuracy of the sparse training model fluctuates greatly, but the accuracy then increases as the training process continues and the learning rate is fine-tuned. Though the accuracy is slightly inferior to that of normal training, sparse training can detect less important channels and thus perform network pruning wisely, which effectively reduces the weight of the network.

**Figure 10:**
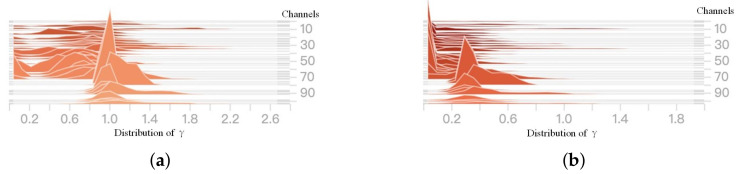
Comparison of gamma distribution before and after training. (**a**) Before sparse training. (**b**) After sparse training.

The scale factor of BN was used to select the channels with lower importance for pruning. The distribution of BN before and after sparse training is shown in Figure 10. It can be seen that the output of each layer is Gaussian-like after normal training. However, after sparse training, the outputs of many layers will tend to zero. Therefore, it is proven that the importance of each channel can be effectively distinguished by the scale factor.

The weight of the network influences the network size, the occupied memory size, and the inference speed. In the pruning process, the pruning proportion of the channels also affects all aspects of the network performance. A comparison experiment of the original YOLOv3 and the pruned YOLOv3 was performed under different pruning ratios, as shown in Table 3. From the actual data, it can be seen that the appropriate pruning ratio can not only reduce the network size and thus greatly accelerate the inference speed, but can also ensure that the accuracy of the pruned network is close to that of the original detection network. However, if the pruning ratio is excessively large, although the inference speed can be further increased, the accuracy will drastically deteriorate even when the network is fine-tuned. Through comparisons and analyses, this paper selects the network with 40% pruning as the object detection model for express parcels.

The data in Table 3 are the results of using dynamic weights during sparse training. We set $\lambda_{s}=0.001$ and $\lambda_{f}=0.0005$ in the Equation (2). In order to further prove the validity of the dynamic weight used in Equation (2), we conducted a set of comparative experiments with fixed weights. In this experiment, the fixed weights were set to 0.0005 and 0.001 for training separately. Then, the trained network was pruned to different degrees. Finally, the accuracy and inference time were obtained through testing and compared with previous experiments, as shown in Table 4.

After comparison, with similar detection accuracy, the sparse network with dynamic weight could be pruned to a greater degree, that is, a more lightweight network structure was obtained. It greatly improved the inference speed of the network. Excessive static weight would make the degree of sparseness too large, the network representation ability would be reduced, and the detection accuracy would be affected. However, static weights that are too small will cause the degree of sparsity to not be obvious, which is not conducive to distinguishing the importance of channels. When the pruning ratio is large, the accuracy will also be significantly reduced. Dynamically adjusting the weight of sparse training can make the degree of sparseness of the network moderate and can ensure the network’s representation ability. Therefore, using dynamic weights can effectively improve the detection accuracy and speed of the network.

**Figure 11:**
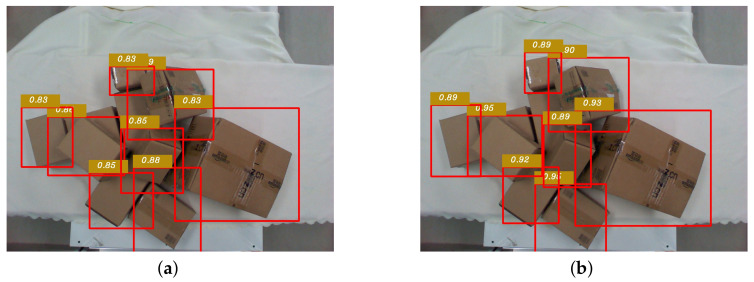
Comparison before and after adding the batch normalization (BN)–rectified linear unit (RELU)–convolution layer (Conv) module. (**a**) Without the BN-RELU-Conv module. (**b**) With the BN-RELU-Conv module.

Another factor affecting the accuracy of detection is that, with the deepening of the network, although the semantic features of the data extracted by the network are richer, a lot of spatial information is lost. The spatial information includes the dependency relationship of each express parcel that blocking others in a disorderly stacked environment. Therefore, by adding the BN–RELU–Conv module to the network, channel features of the shallow feature maps and deep feature maps are fused to extract more delicate features and avoid loss of the spatial information. The experimental results shown in Figure 11 indicate the accuracy of object detection with and without BN–RELU–Conv module. The value in the figure is the confidence level for object recognition.

### 3.2. Experiment on Multi-Task Optimal Sorting Network

**Figure 12:**
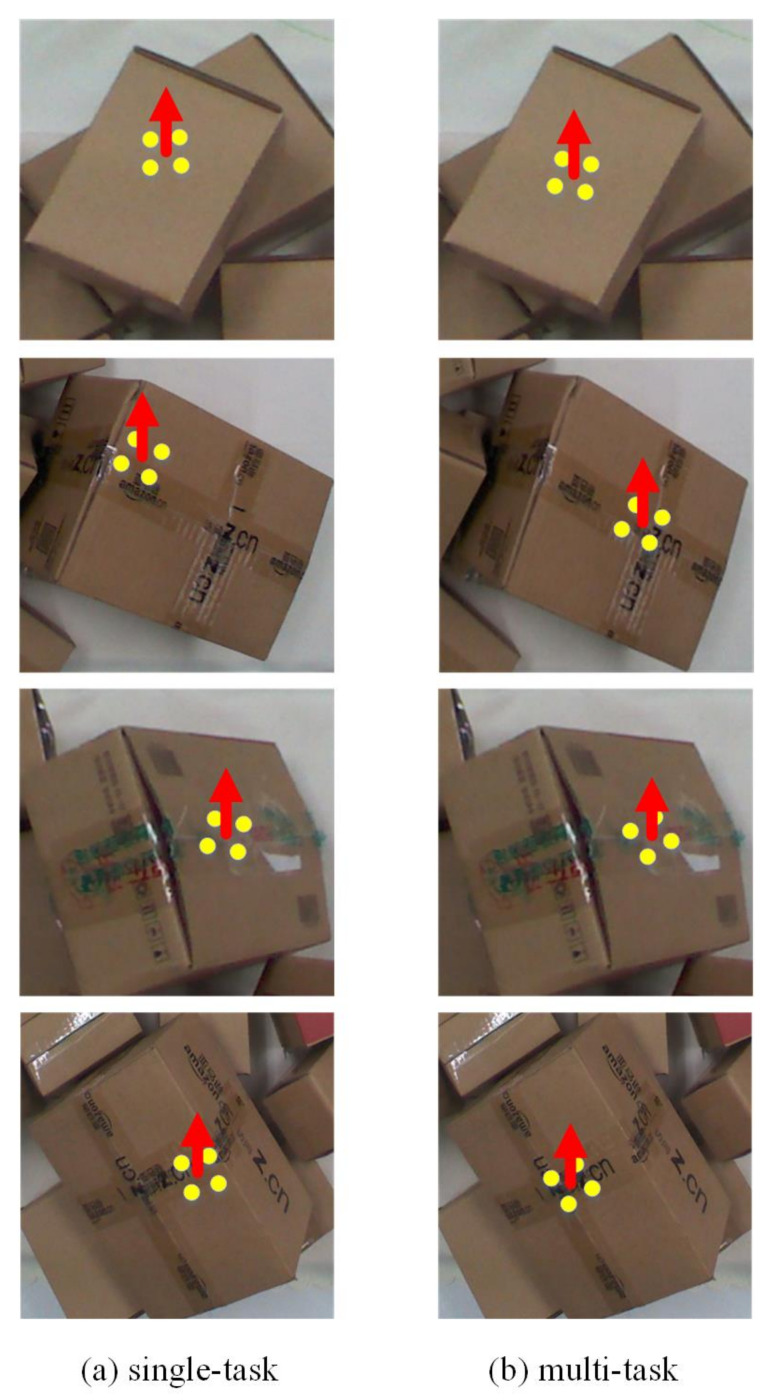
Comparison of the sorting locations of the single-task and multi-task models.

The loss function used by the optimal sorting position and pose estimation network combines the pose estimation bias and the key point position prediction bias, and is trained in parallel by a multi-task network model. After training, the model is used to infer the sorting position and pose of parcels in the test set. For comparison, we used the single-task network model as a baseline that removes the pose estimation branch and keeps only the key point position bias to predict the picking position. It can be seen from Figure 12 that some sorting positions detected by the single-task model were skewed towards the edge of the box, while the positions estimated by the multi-task model basically appeared in the center of the grasping planes of the parcels. The accuracy of the detection results obtained by the multi-task model is higher, which is more conducive for robots in sorting and conducting the following operations more safely and efficiently. This is because the pose information is better integrated during the training of the multi-task model, making the spatial characteristics of the grasping planes and the sorting vectors more obvious. In addition, as can be seen from the experimental results in Table 5, the multi-task model has a great advantage in detection accuracy.

Compared with the cascaded network model [20], the end-to-end (E2E) network structure used in this paper for training and inference can improve the inference speed, and the loss of accuracy can be compensated by the multi-task model structure. In order to prove this point, a detailed comparative experiment was carried out, and the results for the CPU and GPU are shown in Table 5.

It can be seen from Table 5 that the detection accuracy of our multi-task E2E network model is nearly 5% higher than that of the single-task E2E network, while their speed difference is small. Compared with the single-task cascaded network, our network improves the inference speed by 27.94% without losing accuracy. On the other hand, although the multi-task cascaded network model has slightly higher accuracy, its real-time performance is poor. Therefore, under comprehensive comparisons, the multi-task E2E network model proposed in this paper achieved excellent performance in terms of speed and accuracy, further improving the efficiency and real-time performance of the system.

**Figure 13:**
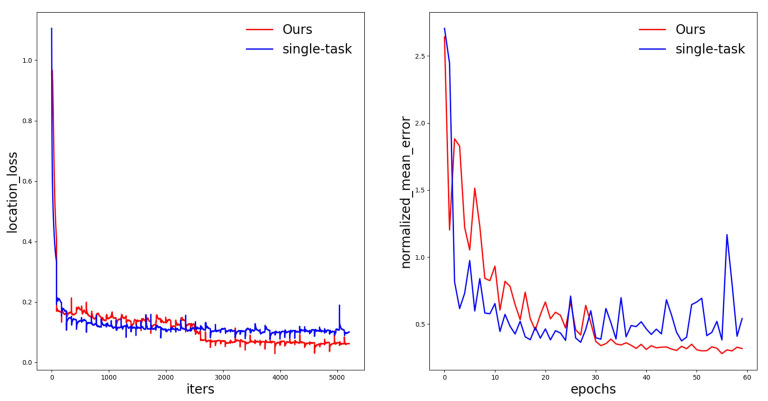
Location loss and normalized mean error (NME) of our model compared with the single-task network model.

In our research, the pose output in the algorithm was used to help fine-tune key point detection. So, we used the obtained position coordinates to get the posture of the express package through Equation (11). The accuracy of the position detection directly affects the accuracy of the pose estimation. The curves in Figure 13 show the location loss and the NME (normalized mean error) of our model during the training process compared with the single-task network model. As can be seen from the figure, the multi-task network structure has a faster convergence speed, a more stable training process, and a more accurate detection accuracy for position and pose estimation.

In order to further prove the superiority of our method, we compare it with some representative solutions [10,14,16,30]. The solutions in [14,16] are based on semantic segmentation and can detect regions that can be grasped by robots. The method in [10,30] directly performs grasp detection using a deep neural network after modeling the robot’s grasping end effector as a vector. Because these methods are different from the application scenario of ours, we select the geometric center of the above detection results as the optimal sorting position for comparison. The results of the experiment are shown in Table 6.

It can be seen from Table 6 that our method uses a reasonable pruning strategy, which makes the network lighter and faster than other solutions, but the accuracy is slightly inferior to the other methods [10,16]. However, benefiting from the improved accuracy of the multi-task model, the result of our solution in the parcel sorting task is also satisfactory.

### 3.3. Robot Sorting Experiment

In order to comprehensively evaluate the effectiveness and robustness of our algorithm, the robot sorting system shown in Figure 8 was used for implementing integrated parcel sorting experiments. By using the “hand–eye calibration” of the robot and the RGB-D camera [31], the position and attitude information of the target objects obtained by the algorithm can be transformed into the robot’s base coordinate system; then, the robot is guided to pick up and place the disorderly stacked express packages.

**Figure 14:**
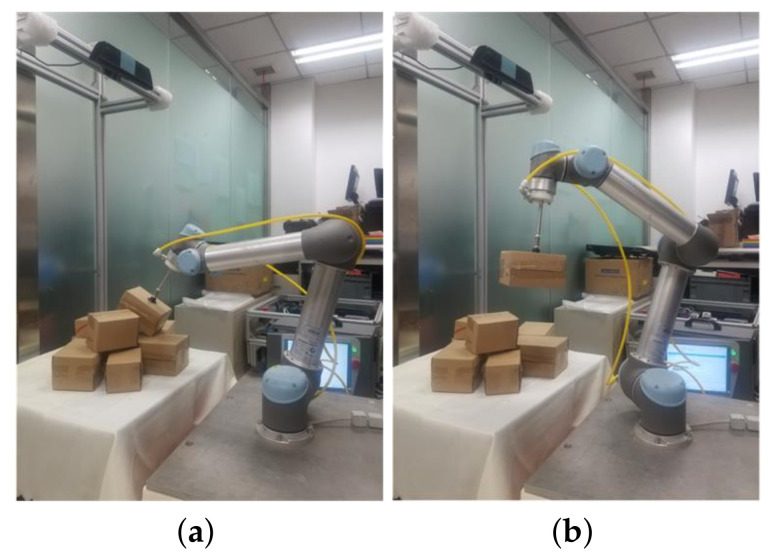

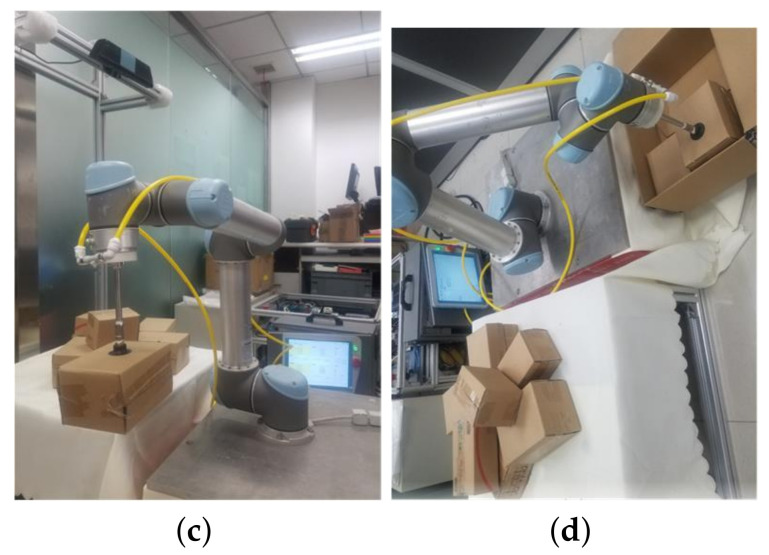
Robot sorting experiment. (**a**) Grasping. **(b**) Picking up. (**c**) Moving. (**d**) Placing.

The experiments were divided into 50 groups with different parcel stacking scenes. The number of stacked parcels in each group of complex scenes was no less than six, that is, the sorting test experiment was performed on more than 300 parcels. The selection process for each package is shown in Figure 14. When all of the parcels in each group of scenes were picked up and placed in the designated positions according to a certain criterion, this group of experiments was completed. After the experiments, the robot sorting system reached a sorting success rate of 89.7%. At the same time, the average detection time for each parcel was reduced by 11.5% compared with the algorithm in [20]. The time for the neural network to detect each scene was within 20 ms, and the time for the robot to sort was about 10 s (each scene in our experiment included 6–7 boxes). The results of the experiment demonstrate that the proposed algorithm can increase the time efficiency of the sorting system while guaranteeing the accuracy and reliability. So, we think that our system can be used as an integral part of an automated warehouse center to replace manpower.

## 4. Conclusions

In this paper, we propose a novel vision-based robot sorting method for logistics environments, which can improve the time efficiency of parcel sorting in complex scenes while ensuring the accuracy and reliability. First, a lightweight object detection network model was proposed, which greatly shortens the detection time and improves the system’s sorting efficiency and real-time performance. Then, a multi-task model for an optimal sorting position and pose estimation network based on key points was proposed.

The experimental results indicate that our solution for express parcel sorting has significantly better real-time performance under the condition of ensuring accuracy compared with other existing methods. In addition, it can effectively solve the problem of sorting cluttered parcels in express storage in real time. The whole system can greatly reduce labor costs and improve the degree of automation in warehouse centers. Since the dataset in this paper only contains square packages, the detection of packages with special-shaped structures is not accurate enough compared with that of some existing solutions. Further research and optimization are still needed. In the future, we will further optimize and implement the method for efficient sorting of more types of express parcels.

## Figures and Tables

**Table 1:** Size of the feature maps and bounding boxes.

Object Detection Feature Map	Size of Feature Map (${\mathrm{pixel}}^{2}$)	Size of the Preset Bounding Box (${\mathrm{pixel}}^{2}$)	Number of Preset Bounding Boxes
feature map1	13 × 13	(116 × 90); (156 × 198); (373 × 326)	13 × 13 × 3
feature map2	26 × 26	(30 × 61); (62 × 45); (59 × 119)	26 × 26 × 3
feature map3	52 × 52	(10 × 13); (16 × 30); (33 × 23)	52 × 52 × 3

**Table 2:** Object detection network hyperparameter setting.

Hyperparameters	Value
batch size	8
base learning rate	0.01
learning rate decay	2000, 4000
rate of learning rate change	0.1
maximum iterations	8000
momentum	0.9
weight decay	0.0005

**Table 3:** Accuracy and speed performance under different pruning ratios.

Model	MAP(%)	Parameter Amount (MB)	Parameter Reduction (%)	Inference Time (s)	Time Reduction (%)
YOLOv3 (Baseline)	98.75	58.67	—	0.0161	—
YOLOv3 (10%Pruned)	97.28	57.76	1.55	0.0158	1.82
YOLOv3 (20%Pruned)	97.25	54.55	7.21	0.0156	3.11
YOLOv3 (40%Pruned)	96.58	38.78	33.9	0.0131	18.63
YOLOv3 (60%Pruned)	65.34	20.67	64.8	0.0103	36.02
YOLOv3 (80%Pruned)	20.26	6.36	89.2	0.0101	37.28

**Table 4:** Accuracy and speed performance using dynamic and fixed weights.

Sparse Weight	Pruning Ratio	MAP (%)	Inference Time (s)
fixed 0.0005	20%	93.43	0.0156
fixed 0.0005	40%	90.84	0.0132
fixed 0.001	20%	91.98	0.0155
fixed 0.001	40%	89.67	0.0131
$\lambda_{d}$ ($\lambda_{s}=0.001$, $\lambda_{f}=0.0005$)	20%	97.25	0.0156
$\lambda_{d}$ ($\lambda_{s}=0.001$, $\lambda_{f}=0.0005$)	40%	96.58	0.0131

**Table 5:** Speed performance comparison for different model structures for the sorting network.

Structure	Detection Accuracy (%)	Inference Time in CPU (s)	Inference Time in GPU (s)
end-to-end + single-task	93.62	0.2572	0.01674
cascaded + single-task	95.86	0.3836	0.02607
cascaded + multi-task	98.34	0.4258	0.03127
end-to-end + multi-task	97.53	0.2764	0.01833

**Table 6:** Comparison of different solutions for parcel sorting tasks.

Method	Inference Time (s)	Detection Accuracy (%)
BB-CNN-CRF [14]	0.3162	96.36
AffordanceNet [16]	0.1508	98.81
FCGD [10]	0.0596	98.31
Grasp detection using Faster RCNN [30]	0.0489	97.44
Ours	0.0183	97.53
